# Ultrasound-Assisted Cerium-Doped Zeolite Nanocomplex Attenuates Adenomyosis via Wnt/β-Catenin Pathway Inhibition and IGFBP5 Downregulation in SFRP4^+^ NKT Cells

**DOI:** 10.33549/physiolres.935738

**Published:** 2026-06-01

**Authors:** Xulin LI, Juan HU, Chong FENG

**Affiliations:** 1 Department of Ultrasound Medicine, Wuxi No. 8 People’s Hospital, Wuxi, Jiangsu, China; 2 Department of Ultrasound Medicine, Ezhou Central Hospital, Ezhou Hubei, China; 3 Department of Ultrasound Medicine, Hongqi Hospital Affiliated to Mudanjiang Medical University, Mudanjiang Heilongjiang, China

**Keywords:** Adenomyosis, Cerium-doped zeolite, Nanomedicine, Wnt/β-catenin pathway, IGFBP5, SFRP4^+^ NKT cells, Inflammation, Fibrosis, Ultrasound-assisted synthesis

## Abstract

Adenomyosis is a chronic gynecological disorder characterized by ectopic endometrial tissue within the myometrium, leading to pelvic pain, menorrhagia, and infertility. Increasing evidence implicates dysregulated Wnt/β-catenin signaling and aberrant activation of SFRP4^+^ natural killer T (NKT) cells in disease progression. This study reports the ultrasound-assisted synthesis of a cerium-doped zeolite nanocomplex (Ce-ZNC) and evaluates its therapeutic efficacy in experimental models of adenomyosis. Ce-ZNC was synthesized *via* an ultrasound-assisted doping approach and characterized using transmission electron microscopy (TEM), X-ray diffraction (XRD), Fourier-transform infrared spectroscopy (FTIR), dynamic light scattering (DLS), and polydispersity index (PDI) analysis. *In vitro* studies were conducted on human peripheral blood mononuclear cell (PBMC)-derived SFRP4^+^ NKT cells. *In vivo* efficacy was evaluated in a murine adenomyosis model. IGFBP5 expression and Wnt/β-catenin signaling components were analyzed by RT-qPCR and Western blotting. Histological and cytokine analyses were performed to assess fibrosis and inflammation. Ce-ZNC exhibited spherical morphology with nanoscale dimensions and good colloidal stability. Treatment resulted in a dose-dependent suppression of IGFBP5 expression and inhibition of Wnt/β-catenin signaling. *In vivo* administration significantly reduced uterine fibrosis, glandular invasion, and inflammatory cytokine levels. These findings suggest that Ce-ZNC exerts immunomodulatory and anti-fibrotic effects in adenomyosis. While promising, further studies are required to elucidate biodistribution, long-term safety, and translational potential in human models.

## Introduction

Adenomyosis is a prevalent gynecological disorder affecting approximately 20 % of women of reproductive age and is defined by the presence of endometrial glands and stroma within the myometrium [1–3]. This condition is associated with chronic pelvic pain, abnormal uterine bleeding, substantially impairing quality of life [4,5]. Despite its clinical significance, the pathophysiology of adenomyosis remains incompletely understood, and current treatment are largely limited to hormonal suppression or surgical intervention [6,7].

Recent evidence highlights the role of dysregulated Wnt/β-catenin signaling and overexpression of insulin-like growth factor-binding protein 5 (IGFBP5) in promoting inflammation and fibrosis in adenomyotic tissues [8,9]. Additionally, immune dysregulation – particularly involving SFRP4^+^ natural killer T (NKT) cells has emerged as a potential contributor to disease progression. Targeting these molecular and immuno-logical pathways may therefore offer novel therapeutic strategies [10,11].

Although murine models provide valuable mechanistic insights, species-specific differences in uterine immune environments present translational challenges [12–14]. Incorporating both murine and human PBMC-derived immune cells enhance the relevance of preclinical findings [15,16].

In this study, we synthesized a cerium-doped zeolite nanocomplex (Ce-ZNC) using an ultrasound-assisted method and evaluated its therapeutic potential in adenomyosis by targeting Wnt/β-catenin signaling and IGFBP5 expression in SFRP4^+^ NKT cells.

## Materials and Methods

### Ethics

All animal procedures were approved by the Institutional Animal Care and Use Committee and conducted in accordance with National Institutes of Health guidelines and ARRIVE reporting standards.

### Materials and apparatus

Zeolite A (Sigma-Aldrich, USA) and cerium nitrate hexahydrate (Ce(NO_3_)_3_·6H_2_O; Sigma-Aldrich) were obtained from commercial suppliers. Characterization was performed using TEM (JEOL JEM-2100, Japan), XRD (Bruker D8 Advance), FTIR spectrometer (PerkinElmer Spectrum Two), and DLS instruments (Zetasizer Nano ZS, Malvern). Biological reagents included Dulbecco’s Modified Eagle Medium (DMEM), fetal bovine serum (FBS), magnetic-activated cell sorting (MACS) kits (Miltenyi Biotec), antibodies (Cell Signaling Technology) for Western blotting, enzyme-linked immunosorbent assay (ELISA) kits, and RT-qPCR reagents (Qiagen) were employed.

### Study design

The study consisted of two phases: (i) synthesis and physicochemical characterization of Ce-ZNC and (ii) *in vitro* and *in vivo* evaluation of its biological effects in adenomyosis models.

### Animals

Thirty female C57BL/6 mice (6–8 weeks old) were randomly assigned to control, adenomyosis, or adenomyosis + Ce-ZNC groups (n=10/group). Adenomyosis was induced *via* neonatal tamoxifen administration. Ce-ZNC was administered intraperitoneally at 10 mg/kg/week for four weeks [17].

### Synthesis of Ce-ZNC nanocomplex from zeolite A

Zeolite A (1 g) was dispersed in deionized water and mixed with cerium nitrate solution, followed by ultrasonic irradiation (20 kHz, 200 W, 30 min). The product was centrifuged, washed, dried, and calcined at 450 °C [18].

### Characterization

Nanoparticles were characterized by TEM, XRD, FTIR, DLS, and PDI measurements. All analyses were performed in triplicate.

### Cell isolation and in vitro assays

Human PBMCs were isolated by Ficoll density gradient centrifugation. SFRP4^+^ NKT cells were purified using MACS and treated with Ce-ZNC (0–100 μg/ml). IGFBP5 expression was quantified by RT-qPCR. Western blotting assessed β-catenin, cyclin D1, and phosphorylated GSK-3β levels, normalized to β-actin [19,20].

### Histological and cytokine analysis

Uterine tissues were stained with hematoxylin and eosin (H&E) and Masson’s trichrome. Fibrosis was scored by blinded pathologists. IL-6 and TNF-α levels were quantified by ELISA [17,19].

### Statistical analysis

Data were analyzed using one-way ANOVA with Tukey’s *post hoc* test. Results are expressed as mean ± SD, with p<0.05 considered significant.

## Results

### Nanocomplex characterization

**Fig. 1:**
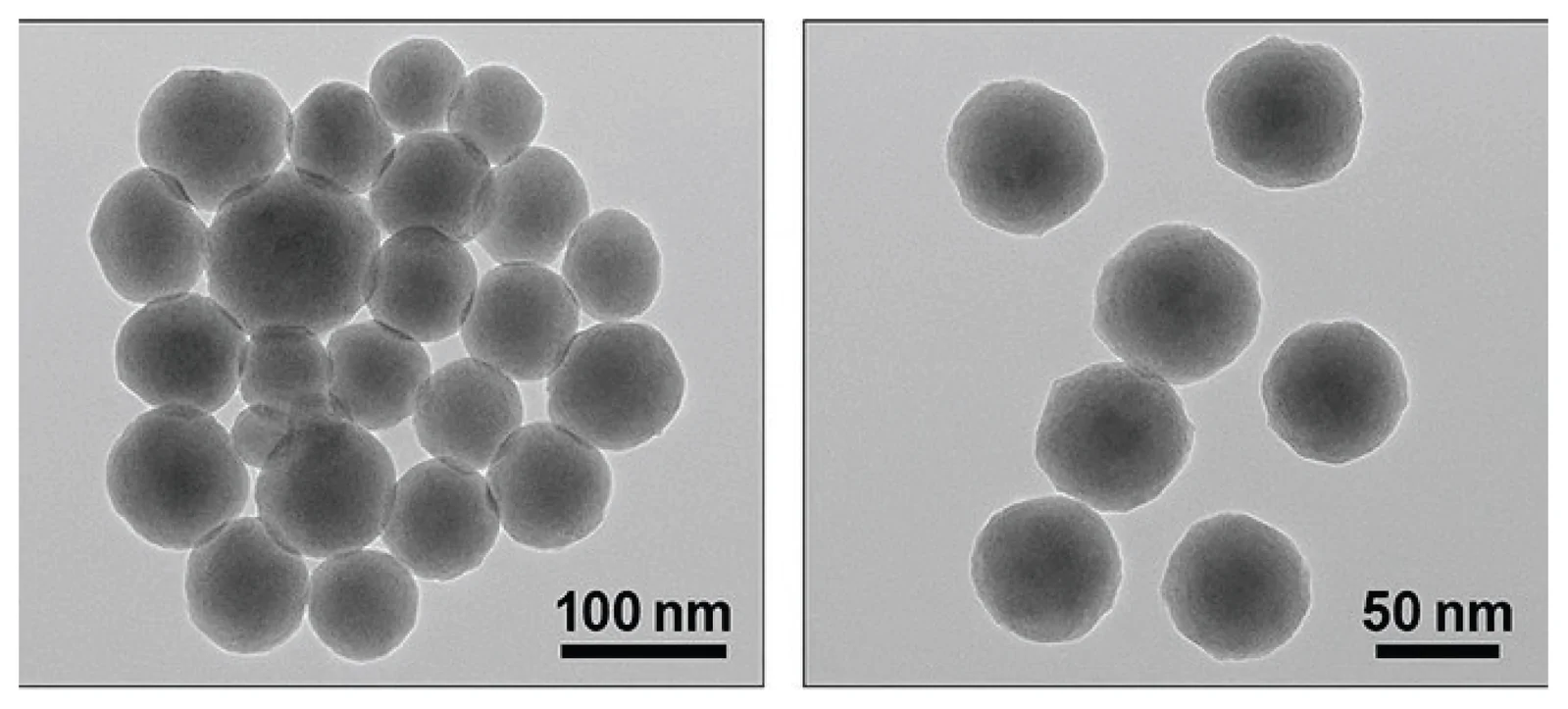
Transmission electron microscopy (TEM) image of cerium-doped zeolite nanocomplex (Ce-ZNC). Representative TEM micrograph showing spherical Ce-ZNC nanoparticles with a uniform distribution and average diameters ranging from 50 to 100 nm. The image confirms successful cerium doping into the zeolite matrix.

Ce-ZNC particles were spherical with diameters of 50–100 nm. XRD confirmed cerium incorporation, FTIR identified characteristic Si-O and Ce-O bonds, and DLS showed a mean hydrodynamic diameter of 92.3±5.1 nm with a PDI of 0.21, indicating uniform size distribution. Figure 1 shows a representative TEM micro-graph demonstrating the spherical morphology and nanoscale dimensions of the Ce-ZNC particles.

Table 1 shows a summary of physicochemical properties of Ce-ZNC. This table presents the key structural and physicochemical parameters of the synthesized Ce-ZNC nanocomplex, confirming its spherical morphology, nanoscale size, successful cerium incorporation, and surface stability characteristics essential for biological applications.

### Suppression of IGFBP5 expression

Ce-ZNC induced a dose-dependent reduction in IGFBP5 mRNA levels in SFRP4^+^ NKT cells, with up to 79 % suppression at 100 μg/ml (p<0.01) (Table 2). Table 2 shows a IGFBP5 gene expression in SFRP4^+^ NKT cells treated with Ce-ZNC. This Table displays the quantitative PCR results showing a clear dose-dependent reduction in IGFBP5 mRNA levels upon Ce-ZNC treatment, indicating its effectiveness in gene expression modulation relevant to fibrotic signaling.

Table 2 shows a IGFBP5 gene expression in SFRP4^+^ NKT cells treated with Ce-ZNC. This Table displays the quantitative PCR results showing a clear dose-dependent reduction in IGFBP5 mRNA levels upon Ce-ZNC treatment, indicating its effectiveness in gene expression modulation relevant to fibrotic signaling.

### Inhibition of Wnt/β-Catenin signaling

**Fig. 2:**
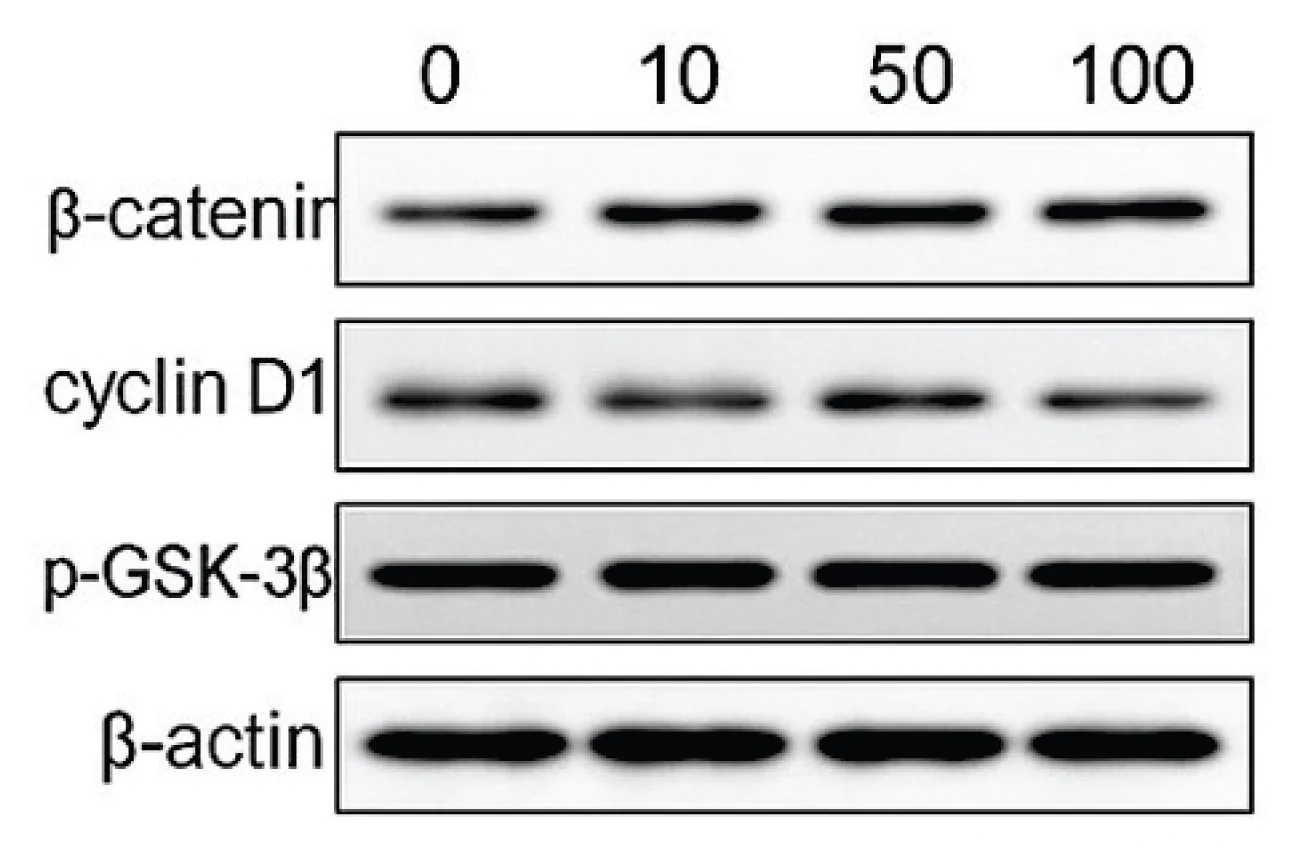
Inhibition of Wnt/β-catenin signaling.

Western blot analysis demonstrated decreased β-catenin and cyclin D1 expression and increased GSK-3β phosphorylation following Ce-ZNC treatment (Fig. 2). Western blot analysis revealed a corresponding decrease in β-catenin and cyclin D1 protein levels, along with an increase in phosphorylated GSK-3β (p-GSK-3β), indicating inhibition of the Wnt/β-catenin signaling pathway. Figure 2 presents representative Western blot band intensities, confirming the downregulation of β-catenin and cyclin D1 and the increase of phosphorylated GSK-3β.

### In vivo outcomes in mice: Histological and cytokine analysis

**Fig. 3:**
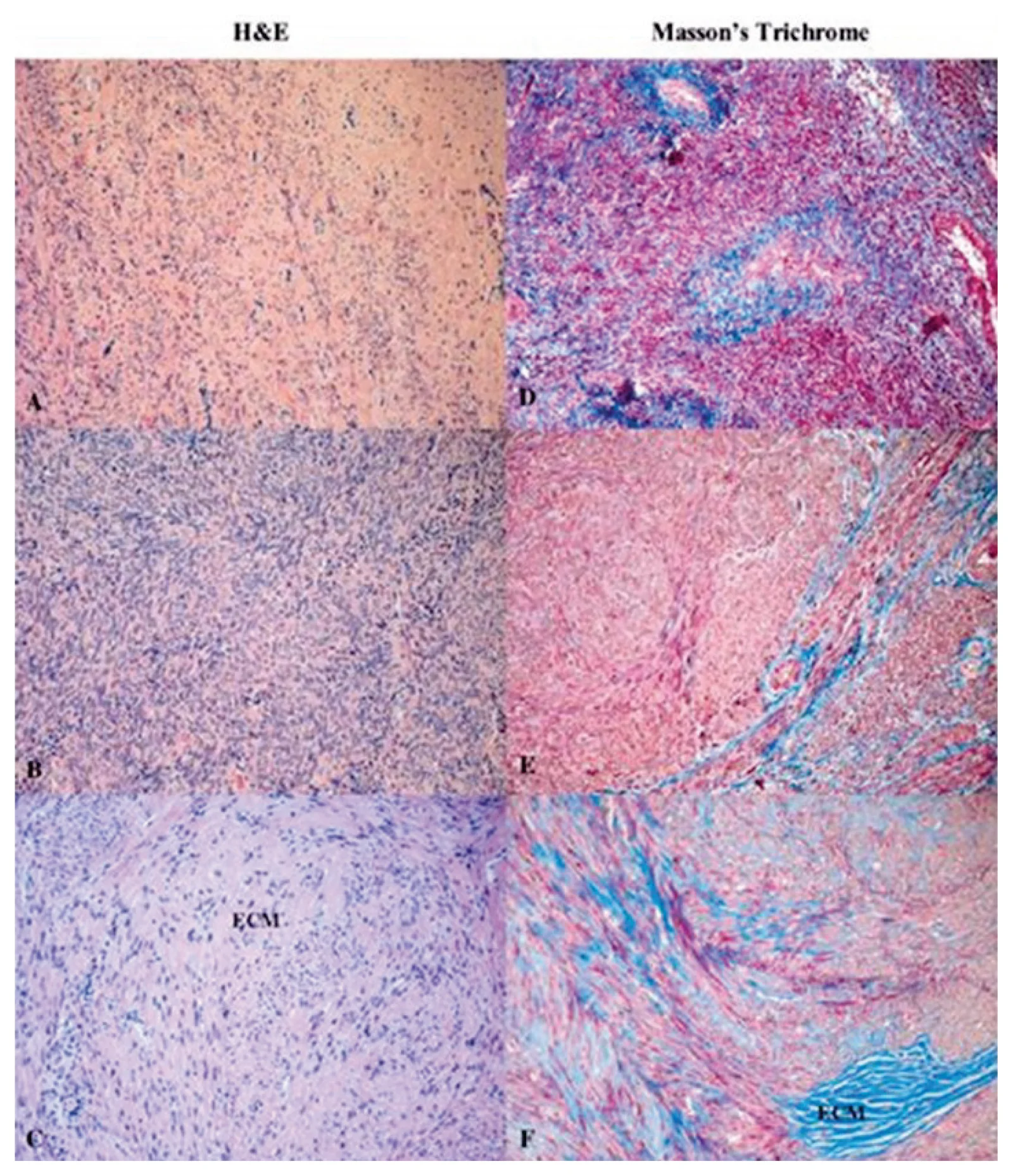
Representative histological images of uterine tissue in control, adenomyosis, and Ce-ZNC-treated mice. Left panels: H&E staining. Right panels: Masson’s trichrome staining, where collagen fibers appear blue and smooth muscle fibers and cells appear red. Reduced blue-stained collagen in Ce-ZNC-treated tissue suggests a decrease in fibrosis compared to untreated adenomyosis.

Histopathological evaluations of uterine tissues using H&E and Masson’s trichrome staining showed clear distinctions between groups. In the adenomyosis group, extensive glandular invasion and muscular disorganization were noted. These pathological features were substantially ameliorated in the Ce-ZNC-treated group. Figure 3 shows representative histological sections stained with H&E and Masson’s trichrome, highlighting the reduction in glandular invasion and collagen deposition following Ce-ZNC treatment.

Ce-ZNC-treated mice showed significantly reduced glandular invasion, collagen deposition, and fibrosis scores compared to untreated adenomyotic mice. Pro-inflammatory cytokines IL-6 and TNF-α were reduced by over 50 %.

Table 3 summarizes the fibrosis severity in uterine tissue samples across different groups, evaluated by pathologists using a standardized scoring system. Ce-ZNC treatment significantly lowered fibrosis scores compared to untreated adenomyotic tissue. Histological staining revealed significant reduction in glandular invasion and fibrosis in Ce-ZNC-treated mice. Fibrosis scores decreased from 3.2±0.4 (adenomyosis group) to 1.1±0.3 (Ce-ZNC group, p<0.001) (Table 3).

**Fig. 4:**
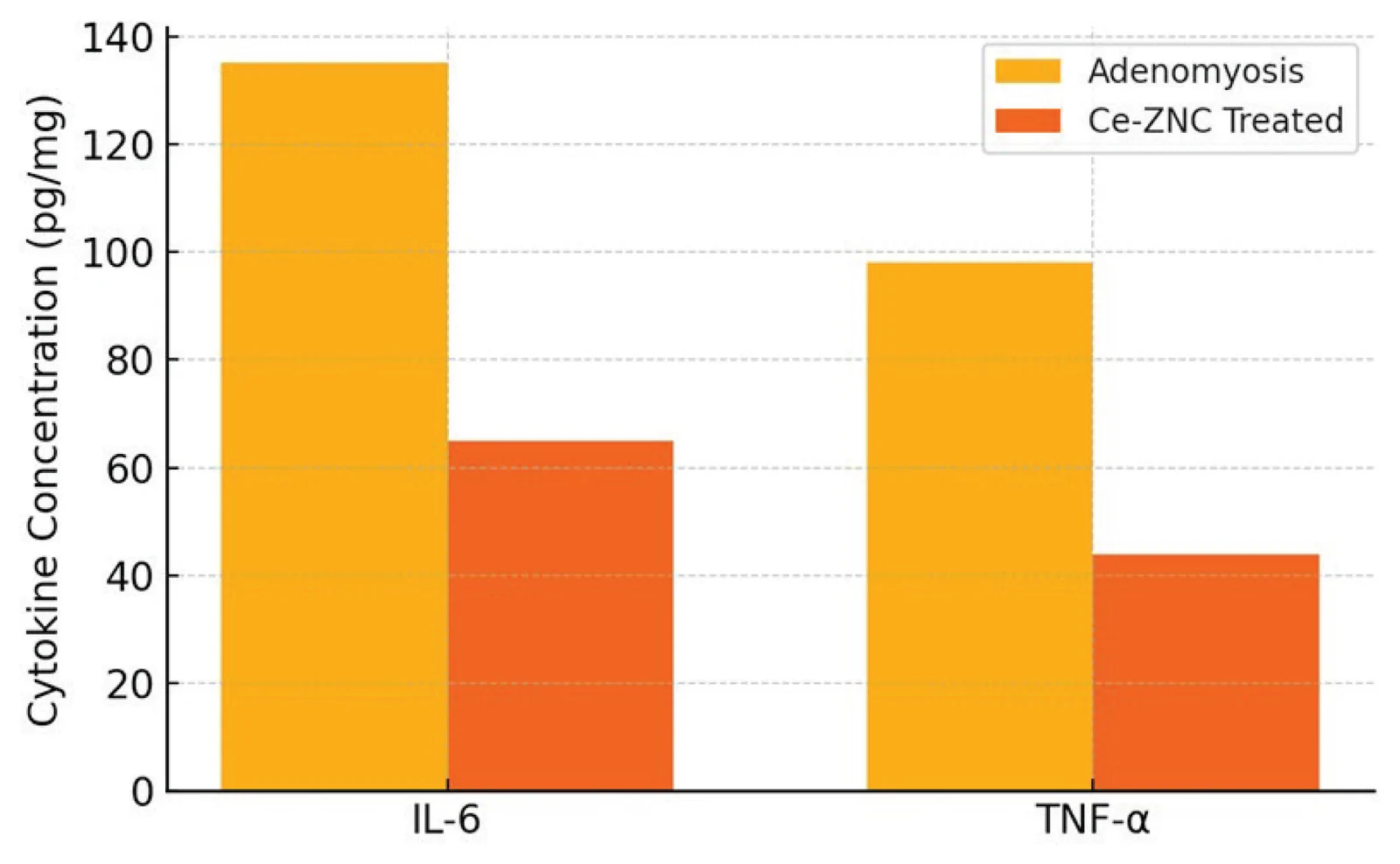
Inflammatory cytokine levels.

ELISA demonstrated reduced IL-6 and TNF-α levels in Ce-ZNC-treated tissues by 51.8 % and 55.1 %, respectively (p<0.05) (Table 4). Measurement of inflammatory cytokines in uterine homogenates revealed a significant decline in both IL-6 and TNF-α levels in the Ce-ZNC-treated group compared to the adenomyosis-only group. These findings corroborate the anti-inflammatory potential of the Ce-ZNC formulation. Table 4 compares the uterine levels of IL-6 and TNF-α among experimental groups. Treatment with Ce-ZNC markedly reduced these pro-inflammatory markers, reflecting the compound’s anti-inflammatory potential. Figure 4 graphically displays the measured concentrations of IL-6 and TNF-α, highlighting significant reductions in the Ce-ZNC treatment group.

## Discussion

This study aimed to. the therapeutic effects of a cerium-doped zeolite nanocomplex (Ce-ZNC) on adenomyosis, with a specific focus on modulating the Wnt/β-catenin signaling pathway and IGFBP5 gene expression in SFRP4^+^ NKT cells. The findings strongly support the initial hypothesis: Ce-ZNC significantly reduced IGFBP5 mRNA expression and downregulated β-catenin signaling components both *in vitro* and *in vivo*, while mitigating histological hallmarks of adenomyosis, including fibrosis and inflammation [9,11].

The reduction in IGFBP5 levels is particularly noteworthy, as IGFBP5 has been implicated in the promotion of fibrosis in endometrial and myometrial tissues. Its suppression *via* Ce-ZNC treatment underscores a promising molecular mechanism for anti-fibrotic intervention. In parallel, the suppression of β-catenin and cyclin D1 and the upregulation of p-GSK-3β suggest that Ce-ZNC acts as a potent inhibitor of canonical Wnt signaling, a pathway previously shown to be upregulated in adenomyotic lesions [8,9,11].

This study demonstrates that Ce-ZNC effectively attenuates adenomyosis-associated inflammation and fibrosis by targeting IGFBP5 expression and Wnt/β-catenin signaling in SFRP4^+^ NKT cells. The dual validation using *in vitro* human immune cells and an *in vivo* murine model strengthens the translational relevance of these findings.

These findings align well with previous literature [8,21]. Some study emphasized the role of β-catenin signaling in myometrial invasion, while another study identified IGFBP5 as a fibrogenic driver in adenomyosis. However, unlike previous studies, our research directly links these pathways to a specific immune cell population – SFRP4^+^ NKT cells – and demonstrates that nanotechnology-based intervention can modulate these pathways [8,21].

A major strength of this study is the dual validation through *in vitro* cellular and *in vivo* murine models, which allowed for robust cross-confirmation of Ce-ZNC’s effects. The ultrasound-assisted synthesis method also ensured the reproducibility and homogeneity of the nanocomplex, contributing to its consistent biological performance [15,22].

Nonetheless, several limitations must be acknowledged. First, the precise mechanism of Ce-ZNC uptake and localization within the uterus and NKT cells remains uncharacterized. Second, long-term safety data and pharmacokinetic profiles were not included and warrant further investigation. Third, although human PBMC-derived NKT cells were used *in vitro*, clinical translation would require testing in human uterine explants or organoid models [8,23].

Although murine models provide valuable mechanistic insights, interspecies differences – especially in uterine immune architecture – necessitate caution when translating findings. Comparative studies indicate that while murine SFRP4^+^ NKT cells share functional markers with their human PBMC-derived counterparts, differences in cytokine milieu and receptor expression profiles warrant validation in human uterine explants or 3D organoids [11,15].

The implications of this work are far-reaching. Ce-ZNC offers a novel, non-hormonal strategy for treating adenomyosis, potentially preserving fertility and minimizing systemic side effects. Future research should explore its effects in combination with existing treatments, evaluate alternative doping agents for zeolites, and test its utility in other gynecological fibrotic disorders such as endometriosis or uterine fibroids [23,24].

While Ce-ZNC demonstrated potent effects on SFRP4^+^ NKT cells, the mechanism of cellular uptake remains to be elucidated. Future studies incorporating nanoparticle tracking (e.g., immunofluorescence co-localization, ICP-MS quantification, or fluorescence-labeled Ce-ZNC) are necessary to assess selective biodistribution and intracellular localization within uterine tissues [22,23].

This study did not assess chronic toxicity, systemic clearance, or potential off-target accumulation of Ce-ZNC. Long-term safety remains a critical consideration for clinical translation and should be addressed *via* extended biodistribution and pharmacokinetic studies, potentially involving ICP-MS or radiolabeling methods [23].

From a clinical perspective, ultrasound-assisted synthesis offers scalability and reproducibility compatible with good manufacturing practice (GMP) standards. Ce-ZNC may serve as a non-hormonal or adjunct therapy alongside existing hormonal treatments, potentially reducing systemic side effects and preserving fertility. However, challenges related to dosing optimization, biodistribution, and long-term safety must be addressed prior to clinical translation.

Limitations include the absence of pharmacokinetic and chronic toxicity data, as well as reliance on murine models. Future studies should incorporate human uterine explants or organoid systems and advanced nanoparticle tracking techniques to further validate therapeutic potential.

In summary, this study demonstrates that Ce-ZNC exerts potent immunomodulatory and anti-fibrotic effects by targeting key molecular pathways in adenomyosis, representing a promising direction for nanomedicine-based gynecological therapeutics.

## Conclusions

Ce-ZNC synthesized *via* an ultrasound-assisted approach demonstrates significant immunomodulatory and anti-fibrotic effects in experimental adenomyosis by suppressing IGFBP5 expression and inhibiting Wnt/β-catenin signaling in SFRP4^+^ NKT cells. These findings support Ce-ZNC as a promising nanomedicine-based, non-hormonal therapeutic candidate with potential for low systemic toxicity. Further translational and safety studies are warranted to advance this approach toward clinical application.

## Figures and Tables

**Table 1:** Summary of physicochemical properties of Ce-ZNC.

Parameter	Observation
*TEM*	Spherical nanoparticles (50–100 nm)
*XRD*	Characteristic zeolite peaks + CeO_2_ peaks
*FTIR*	Bands at 460, 555, and 1000 cm^−1^ (Si-O, Ce-O)
*DLS*	Mean size: 92.3 ± 5.1 nm; Zeta: −18.5 ± 1.7 mV

**Table 2:** FBP5 expression in SFRP4+ NKT cells following Ce-ZNC treatment.

Ce-ZNC (μg/ml)	IGFBP5 Relative Expression (Mean ± SD)
*0 (Control)*	1.00 ± 0.09
*10*	0.68 ± 0.07 (p<0.05)
*50*	0.39 ± 0.05 (p<0.01)
*100*	0.21 ± 0.04 (p<0.01)

**Table 3:** Fibrosis scores (semi-quantitative scale 0–4).

Group	Fibrosis Score (Mean ± SD)
*Control*	0.5 ± 0.2
*Adenomyosis*	3.2 ± 0.4
*Adenomyosis + Ce-ZNC*	1.1 ± 0.3 (p<0.001)

**Table 4:** Inflammatory cytokine concentrations.

Cytokine	Adenomyosis (pg/mg)	Ce-ZNC Treated (pg/mg)	% Reduction
*IL-6*	135 ± 12	65 ± 9	51.8 %
*TNF-α*	98 ± 10	44 ± 8	55.1 %

All findings were statistically significant (p<0.05 or better) and consistent across biological replicates (n=10 mice/group, 3 technical replicates/sample).
